# Supplementary material for: A fault-intrusion-tolerant system and deadline-aware algorithm for scheduling scientific workflow in the cloud
Source: PeerJ Comput Sci. 2021 Nov 2;7:e747. doi: 10.7717/peerj-cs.747 (PMC8576554; doi:10.7717/peerj-cs.747)
Supplement: Supplemental Information 6 [file peerj-cs-07-747-s006.zip › Myproject1.1/docs/org/cloudbus/cloudsim/examples/power/random/package-tree.html]

org.cloudbus.cloudsim.examples.power.random Class Hierarchy


JavaScript is disabled on your browser.


- Overview
- Package
- Class
- Use
- Tree
- Deprecated
- Index
- Help

- Prev
- Next

- Frames
- No Frames

- All Classes

# Hierarchy For Package org.cloudbus.cloudsim.examples.power.random

Package Hierarchies:

- All Packages

## Class Hierarchy

- java.lang.Object
  - org.cloudbus.cloudsim.examples.power.random.Dvfs
  - org.cloudbus.cloudsim.examples.power.random.IqrMc
  - org.cloudbus.cloudsim.examples.power.random.IqrMmt
  - org.cloudbus.cloudsim.examples.power.random.IqrMu
  - org.cloudbus.cloudsim.examples.power.random.IqrRs
  - org.cloudbus.cloudsim.examples.power.random.LrMc
  - org.cloudbus.cloudsim.examples.power.random.LrMmt
  - org.cloudbus.cloudsim.examples.power.random.LrMu
  - org.cloudbus.cloudsim.examples.power.random.LrrMc
  - org.cloudbus.cloudsim.examples.power.random.LrrMmt
  - org.cloudbus.cloudsim.examples.power.random.LrrMu
  - org.cloudbus.cloudsim.examples.power.random.LrrRs
  - org.cloudbus.cloudsim.examples.power.random.LrRs
  - org.cloudbus.cloudsim.examples.power.random.MadMc
  - org.cloudbus.cloudsim.examples.power.random.MadMmt
  - org.cloudbus.cloudsim.examples.power.random.MadMu
  - org.cloudbus.cloudsim.examples.power.random.MadRs
  - org.cloudbus.cloudsim.examples.power.random.NonPowerAware
  - org.cloudbus.cloudsim.examples.power.random.RandomConstants
  - org.cloudbus.cloudsim.examples.power.random.RandomHelper
  - org.cloudbus.cloudsim.examples.power.RunnerAbstract
    - org.cloudbus.cloudsim.examples.power.random.RandomRunner
  - org.cloudbus.cloudsim.examples.power.random.ThrMc
  - org.cloudbus.cloudsim.examples.power.random.ThrMmt
  - org.cloudbus.cloudsim.examples.power.random.ThrMu
  - org.cloudbus.cloudsim.examples.power.random.ThrRs

- Overview
- Package
- Class
- Use
- Tree
- Deprecated
- Index
- Help

- Prev
- Next

- Frames
- No Frames

- All Classes
